# Emotional Competences of Primary Education Teachers: A Need in School Post COVID-19

**DOI:** 10.3390/ejihpe13100141

**Published:** 2023-09-22

**Authors:** Roberto Sanz-Ponce, Elena López-Luján, Ángela Serrano-Sarmiento, Juan Antonio Giménez-Beut

**Affiliations:** 1 Department of General Didactics, Theory of Education and Technological Innovation, Catholic University of Valencia, 46110 Valencia, Spain; elena.lopez@ucv.es (E.L.-L.); jantonio.gimenez@ucv.es (J.A.G.-B.); 2 Inclusive Education and Socio-Community Development, Catholic University of Valencia, 46110 Valencia, Spain; angela.serrano@ucv.es

**Keywords:** emotional competences, teachers, COVID-19, conflicts, teaching skills

## Abstract

The COVID-19 pandemic has increased the number of students with mental health problems: depression, anxiety, stress. Faced with this reality, teachers and schools must be prepared to respond quickly and effectively. Therefore, the objective of this article is to analyze the emotional competences of primary school teachers in the city of Valencia based on the following sociodemographic variables: sex, age, professional experience, type of center and whether they have children. For this purpose, a quantitative methodological approach has been followed, through which the emotional competencies of primary education teachers are analyzed. These results allow us to establish teacher profiles according to sociodemographic variables and help to detect possible training deficiencies. A sample of 371 teachers of primary education in the city of Valencia has been analyzed. The Questionnaire on Teaching Competences of Primary Education Teachers, carried out under the Planned Action Model, has been used, and descriptive, univariate, bivariate and cluster analyses have been carried out. The mean, the standard deviation and the interquartile range (IQR) have been analyzed, as well as non-parametric tests such as the Wilcoxon, Kruskal–Wallis or Z test. The most significant results are that teachers have a greater ability to interpret emotions and to listen to students. On the contrary, it is teachers who most reject prejudice, discrimination and racism. Younger teachers are the ones who implement more inclusive learning environments. Finally, in general, all teachers are very respectful of students and claim to know how to manage classroom conflicts. The results obtained, in general terms, coincide with most of the research on teachers’ emotional competencies. Some aspects simply do not coincide with the literature. The teachers who participated in our research perceive themselves as having a greater capacity to observe and interpret students’ emotions, to generate learning situations that cater to diversity and to listen to their students. Other studies place these competencies at lower levels.

## 1. Introduction

Are teachers prepared to deal with the emotional problems caused by COVID-19 among their students? Do they have the right professional skills? These questions are necessary today, when many students are in a situation of vulnerability [1]. Since March 2020—the time of school closures—research on teaching competencies has focused on teachers’ ability to manage online education [2,3,4,5] or to compensate for the possible learning gaps caused by this teaching [6,7,8,9,10].

Currently, the question is whether they can address the possible psycho-emotional sequelae detected among students. Confinement, safe distancing, masks, the fear of contagion, the loss of family members and over information in the media have generated stress, depression, anxiety, exhaustion, fear and uncertainty [11], along with relationship problems between peers.

With respect to teachers, it was found that, after COVID, those with long experience and high levels of compassion suffered to a greater extent [12]. Other studies analyzing the growth trajectories of the levels of psychological well-being of teachers [13] have shown that it decreases significantly in those who are older and more experienced, affecting positive relationships with others.

UNESCO [14] already called for the inclusion of socio-emotional competencies and resilience in teacher training to care for both students and teachers to respond to future challenges. It also calls for the development of “emotional resilience” in online teaching and in the return to face-to-face school, as well as promoting the learning of socio-emotional skills among students, to face the future in a positive way [15,16]. In this process, the skills of school leaders that reinforce the socioemotional capacities of educators in their schools are important [17].

Save de Children [18], meanwhile, warned of the risk of disaffection among students with school and the loss of interest in learning due to confinement. Teachers were called out to work affectively, incorporating accompaniment, listening and personalized teaching, to make them a key factor of protection, a “tutor of resilience” [19].

The World Bank [20] also warned of possible mental health problems among students and schools and the need to implement social or emotional learning programs to combat anxiety, stress and low self-esteem and to create positive emotional climates in the classroom [21].

In summary, UNESCO [22] saw the crisis as an opportunity to rethink the meaning of education, review evaluation techniques and instruments, improve the teaching of higher thinking skills (questioning, creativity and problem solving) and socio-emotional skills (empathy, teamwork, collaboration, resilience, proactivity, initiative and responsible behavior) and re-emphasizing the role of school as a safe environment for peer relationships [23].

This article has the following objective. Analyze the emotional competences of teachers of primary education of the city of Valencia according to the following sociodemographic variables: sex, age, professional experience, type of center and whether they have children. These results allow for the establishment of teacher profiles based on sociodemographic variables and help to detect possible training deficiencies. These emotional competences of teachers are very necessary nowadays, due to the consequences and psycho-emotional and mental health problems derived from COVID-19 [24,25,26,27,28] among primary school students: anxiety, stress, depression or fear.

For this purpose, this research proposes two hypotheses to analyze the emotional competences of teachers. These have been described based on the observation of reality, the reading of the scientific literature and the analysis of the results of similar research [29,30,31,32,33,34,35].

**Hypothesis** **1.** *The personal characteristics and emotional competences of teachers interfere in the management of students, finding differences depending on sociodemographic and professional variables (sex, age, professional experience, type of center in which they work, whether they have children and teaching vocation)*.

**Hypothesis** **2.** *Classroom management presents significant differences with respect to the emotional competences of teachers, depending on professional and sociodemographic variables (sex, age, professional experience, type of center in which they work, whether they have children and teaching vocation)*.

This research is part of a broader design to evaluate the teaching competencies of teachers based on the theory of reasoned action of Ajzen and Fishbein [36]. For this purpose, some items were taken from the subscale of beliefs and attitudes of the CCPES II questionnaire [37], which allows for the evaluation of the teacher’s self-perceived socioemotional competencies, such as conflict resolution, active listening, empathy, assertiveness, teaching commitment to students, etc. Teachers’ emotional competencies can be defined as the set of beliefs, attitudes, skills, subjective norms, behavioral intentions and behaviors that favor the adequate management of students and the classroom, taking into account the emotional aspects and feelings of students, teachers and families [30].

## 2. Emotional Competence in Teachers

The teacher is the fundamental piece in the construction of the personal identity of their students, fundamentally their emotional influence [38]. It could even be affirmed that the internal socioemotional competence of teachers is a predictor of the relationship between them and their students [39]. In this process, the influence of teachers’ own identity and their conceptions of power has been demonstrated [40]. It has also been shown that those teachers who have a high emotional competence also have it in their personal well-being and their assertive style, which correlates with educational style [41] and influences learning and the best disposition to learn of the students [42]. For this reason, it is necessary to implement emotional competence in their training [43], since it has a direct impact on the personal, social and academic development of the students (in personal relationships, in the classroom climate, in methodology, in motivation, in academic results, in conflict management, in their self-esteem, empathy or resilience) [44].

### 2.1. Competences for the Management of Students

The scientific literature manifests the link between the emotional competences of teachers and their ability to manage students [45,46,47]. A faculty that does not possess a formed emotional competence cannot educate its students [48]. In addition, there must be an adequate interrelation between cognitive and emotional processes to respect students in their personalized integral development process [49]. Teachers must self-regulate to help students manage their own emotions and interpret them, fostering their self-esteem and enabling them to respond appropriately to life’s challenges [50]. This emotional component is reflected in their teaching style [51], which must be linked to their teaching vocation and their professional identity, which has been shaped during their training [48] and their internship [52].

Hence the importance of university training for future teachers. When comparing the levels of emotional intelligence and empathy among students of different degrees, it was observed that in the first course, the level of emotional attention was higher in education students. And in the fourth year, the levels of emotional repair and clarity increased significantly in the education and medicine degrees [53]. Regarding the differences between university students in early childhood and primary education, no differences have been found in their emotional competencies [34]. The combination of experience and specific training has been shown to be the most important for the majority of the components of emotional competence and the one that best enables students to develop it [32].

### 2.2. Competences for Classroom Management

Several studies show how the emotional competences of teachers impact the climate and management of the classroom and generate an impact on the state of well-being of students [54]. And when practicing teachers are analyzed, it is observed that they perceive themselves as having an average level in this competence. But as they advance in age and experience, they decrease in attention and emotional clarity [33]. In addition, Palomera et al. [55] and Gutiérrez and Buitrago [56] add that contextual and methodological variables must also be considered for conflict management in such a way that having a well-planned didactic style or taking care of the contexts generating adequate learning environments facilitates the classroom climate and reduces the impact of interpersonal conflicts [57]. The emotional competencies of teachers and their values are present in the form of conflict resolution in the classroom, as well as their predisposition to commitment, listening and time for their intervention [58]. On the other hand, it is necessary to create a living, innovative, flexible, dynamic, versatile, changing and transformative learning environment, with a clear didactic intention [59], i.e., an environment that responds to the what, how and why of teaching, as well as one that is based on a deep knowledge of the evolutionary psychology and personal characteristics of students [60], an environment that generates human relationships based on emotions and feelings [61].

## 3. Materials and Methods

This research follows a quantitative methodological approach, through which the emotional competences of primary education teachers are analyzed. 

### 3.1. Participants

The sample has been obtained via non-probability sampling for convenience. Teachers participate voluntarily and anonymously, through the online response of the Questionnaire on Teaching Competences. This was sent to schools through electronic mail. A total of 371 teachers participated, both from public and subsidized schools (Table 1). To avoid bias in the selection, the formula was used for samples of finite populations, with a confidence level of 95% and an estimation error of 5%.

### 3.2. Instrument

The Questionnaire on Teaching Competences of Primary Education Teachers [36], carried out under the Planned Action Model [37], is used. This theory allows us to predict teacher behaviors based on their beliefs, attitudes, skills, subjective norms and behavioral intentions. A discussion group and a commission of experts in primary education put forward the first formulation of the questionnaire items, and all the proposals were submitted to evaluation and judgement. Subsequently, a pilot study was carried out to validate the questionnaire and to debug those items that could present problems (n = 154). During this process, we went from the initial 97 proposals to a pilot questionnaire made up of 65 items, until reaching a final questionnaire made up of 60 items, on a Likert scale with 5 responses, where 1 was Totally Disagree and 5 was Totally Agree. The questionnaire was divided into 6 factors, following the theory of planned action, and its reliability (Cronbach’s alpha = 0.917) and validity (KMO = 0.757) were excellent. For this research, 13 items referring to teachers’ emotional competencies were used (Table 2). The reality resulting from the COVID-19 pandemic, in which a multitude of primary school students present some psycho-emotional health problems, requires the evaluation of teaching skills to face this new challenge.

### 3.3. Data Analysis

The scale (summative) is configured under the criterion of “The more the better.” Thus, the higher the value on the scale obtained through adding up all the scores of the questions, the higher the rating in socioemotional competence (Table 2).

Its evaluative character appears in the right column: CMM indicates a higher score and better rating on the scale, and CMP indicates a higher score and worse rating. These are items presented in a negative way, so for their analysis, the answers have been transformed. Answer 1 equals 5, answer 2 equals 4 and answer 3 equals 3. 

Descriptive, univariate, bivariate and cluster analyses were performed to answer the research questions. In some cases, we used the mean, standard deviation (sd), interquartile range (IQR), minimum, first quartile, median, third quartile and maximum when the type of variable required it (scale variable). 

It is necessary to resort to the corresponding nonparametric tests, the Mann–Whitney–Wilcoxon test, hereinafter the Wilcoxon test, to compare two groups and the Kruskal–Wallis test to compare more than two. For the contrast of proportions, the Z test is used whenever possible for two samples; its objective is to determine if the two independent samples were taken from two populations, which present the same proportion of elements with a certain characteristic. When necessary, the 95% confidence interval is also presented for the proportion of elements that have a certain characteristic. And for the independence analysis for contingency tables, test *χ*^2^ is used, if it is not applicable to Fisher’s exact test. For the analysis of the results, the program R is used, and the s RCommander libraries, the graphical library ggplot2 and the library ca are used for the analysis of correspondences. For some independence contrasts, the RcmdrPlugin.IPSUR library is used [62].

## 4. Results

### Analysis of Compliance with the Hypotheses Raised

**Hypothesis** **1.** *The personal characteristics and emotional competences of teachers interfere in the management of students, finding differences depending on sociodemographic and professional variables (sex, age, professional experience, type of center in which they work, whether they have children and teaching vocation)*.

(a) Ability to interpret emotions

**Figure 1:**
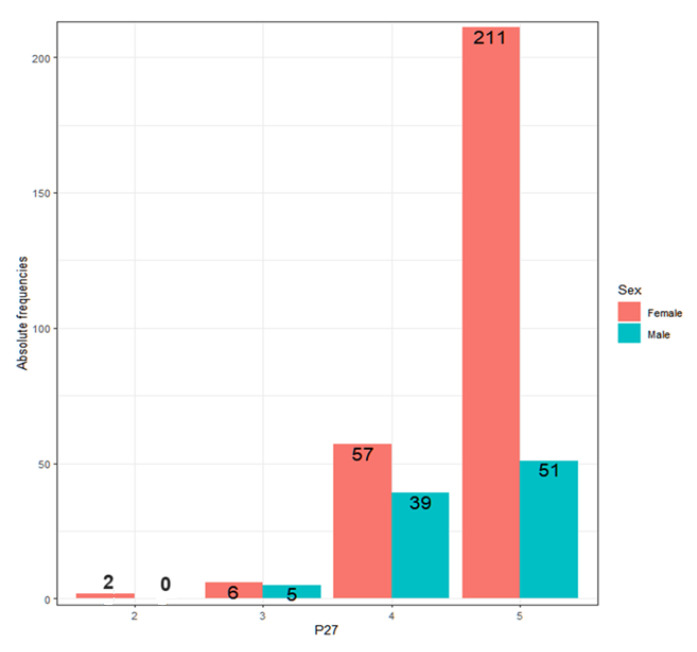
Absolute frequencies according to sex.

**Figure 2:**
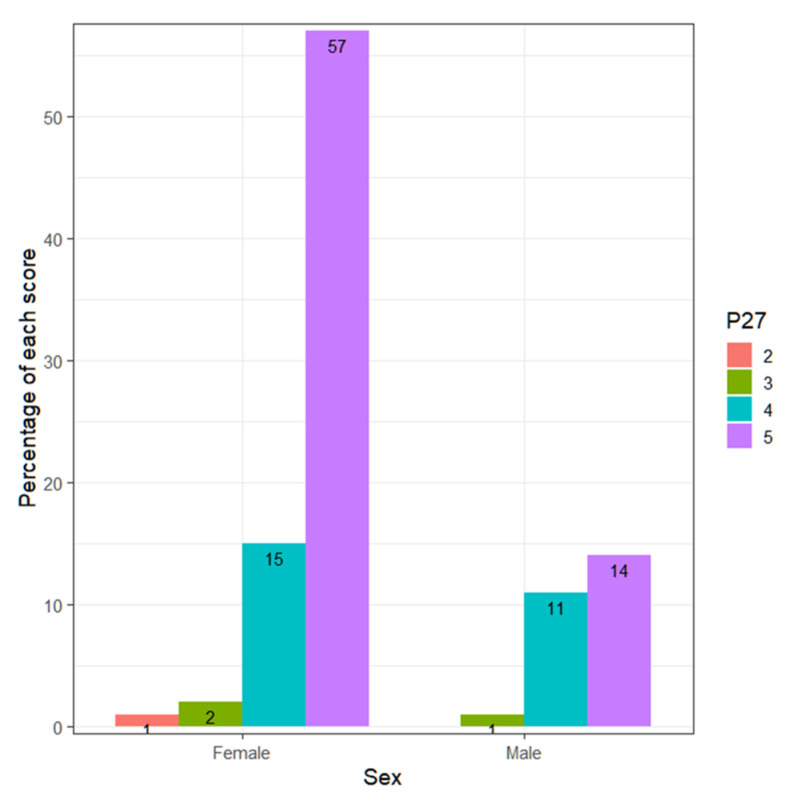
Percentage of each response over the total, by gender.

If the ability of teachers to interpret emotions according to sex is analyzed, it is observed how teachers obtain better results (Wilcoxon, *p*-value = 0.00001717 < 0.05). They can observe and understand the emotions and feelings of their students better than their fellow teachers (Figure 1 and Figure 2).

It should be noted that the proportion of teachers who score high (4 or 5) is CI95% = (93.9, 98.0), that is, the vast majority believes they are able to interpret and observe the emotions and feelings of students. These high scores do not depend on sex (Fisher, *p*-value = 0.3309), the type of center (Fisher, *p*-value = 0.5726), experience (Fisher, *p*-value = 0.1368) or age (Fisher, *p*-value = 0.3380).

**Figure 3:**
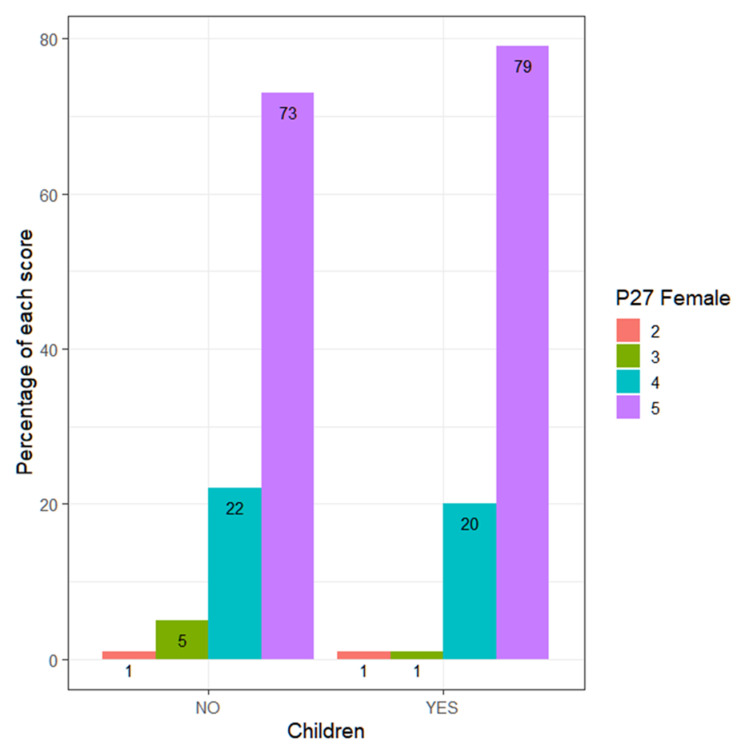
Percentage of each score (women), according to whether they have children or not.

**Figure 4:**
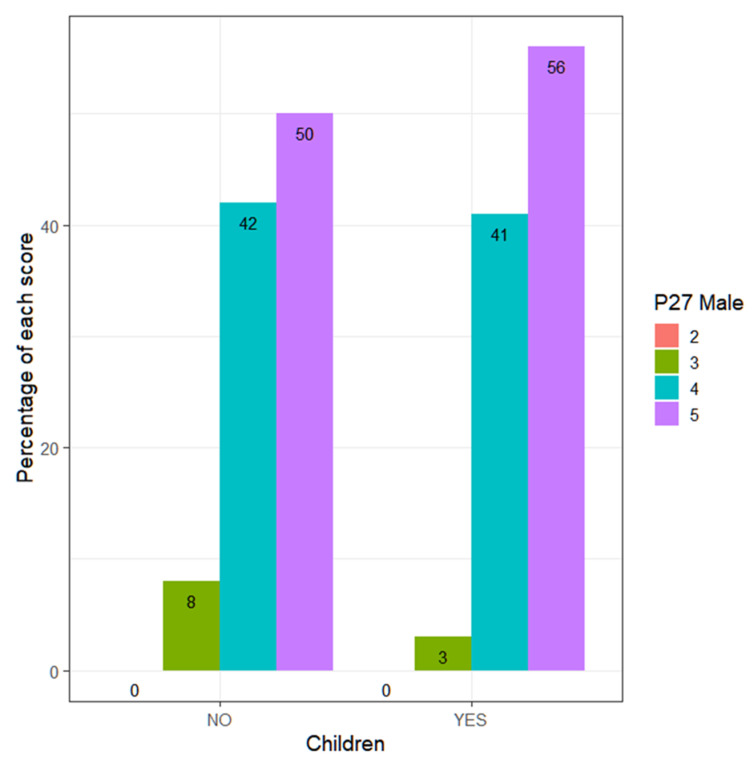
Percentage of each score (men), according to whether they have children or not.

If differences in terms of having children are analyzed, it is observed that for teachers, there are no significant differences (Wilcoxon, *p*-value = 0.2073 > α = 0.05) (Figure 3), nor for teachers (Wilcoxon, *p*-value = 0.4671 > α = 0.05) (Figure 4). This shows that there is no greater sensitivity among teachers with children compared to those without children.

(b) Respect for students

On the other hand, the emotional competencies of teachers also have a lot to do with the way in which teachers interact with students, how they treat them and how they see them. In that sense, it is necessary to analyze the relationship of personal, educational and cultural respects.

**Figure 5:**
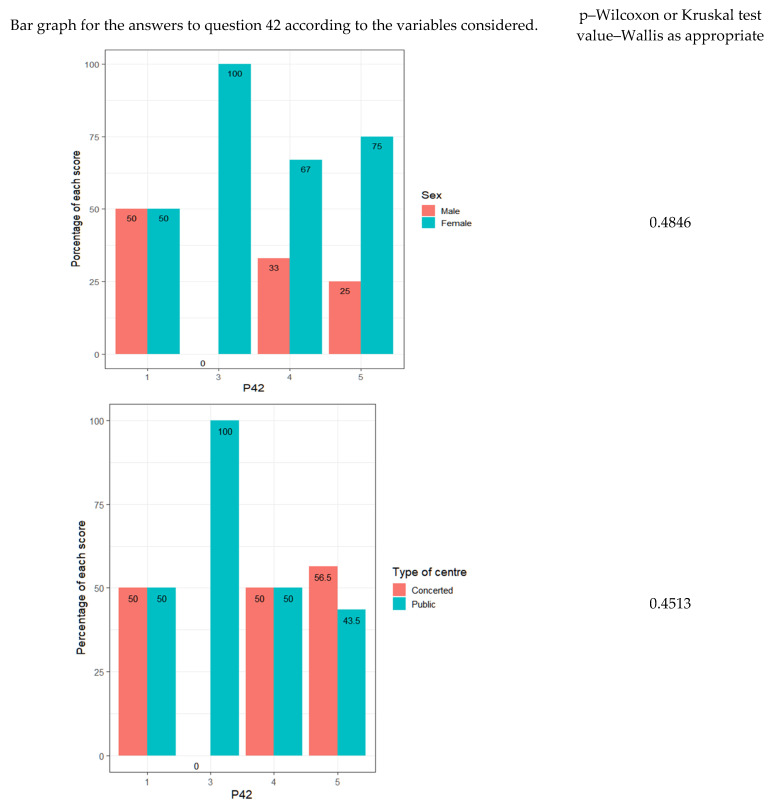

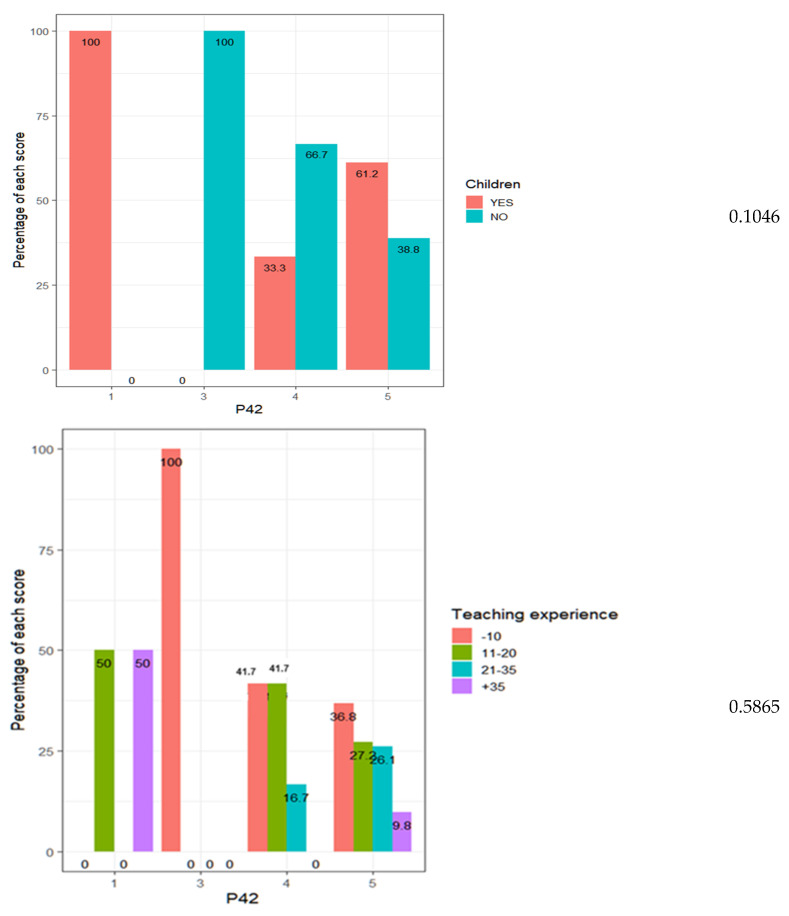

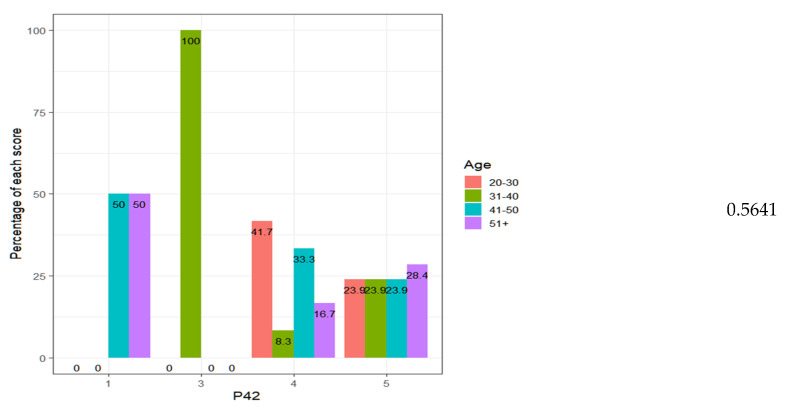
Percentages for each response for sociodemographic variables.

As for whether teachers respect their students, it is observed that the answers do not depend on sex, age, teaching experience, the type of center or whether they have children (Figure 5).

All teachers believe they have an attitude of respect towards students. This positive view is confirmed in the number of teachers who score 4 or 5 (Table 3).

**Figure 6:**
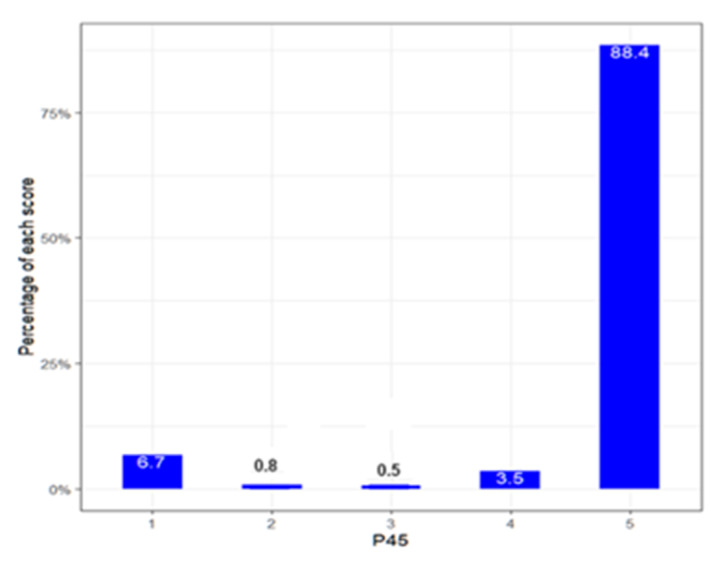
Percentage of responses.

In the same way, teachers present a very positive inclusive vision towards their students. In a very high percentage, they reject prejudice, racism and discrimination, although, unfortunately, there is a percentage of teachers (7.5%) who confess a disrespectful attitude (Figure 6).

**Figure 7:**
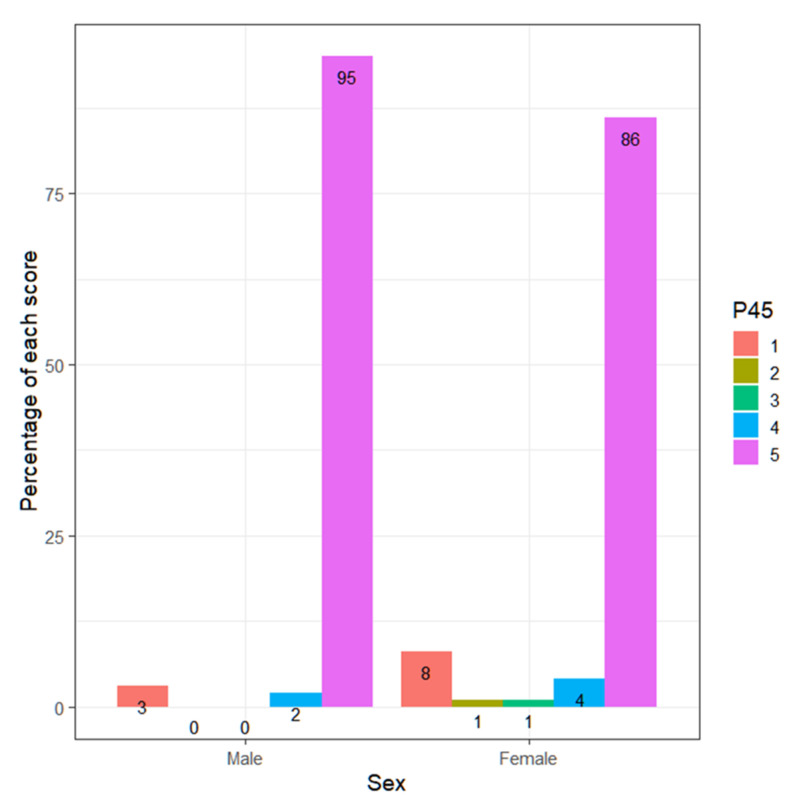
Percentage of each score according to gender.

If analyzed in terms of sociodemographic variables, no differences are observed in terms of age (Kruskal–Wallis, *p*-value = 0.1439 > 0.05), experience (Kruskal–Wallis, *p*-value = 0.6898 > 0.05), the type of center (Wilcoxon, *p*-value = 0.7334 > 0.05) or whether they have children (Wilcoxon, *p*-value = 0.08935 > 0.05, inconclusive). However, there are differences observed regarding sex (Figure 7).

Men score higher than women (Wilcoxon, *p*-value = 0.01283 < 0.05), that is, they reject prejudice, racism and discrimination more. Of the teachers who answer 1, that is, who disagree with the statement of rejecting prejudice, racism and discrimination, 88% are women and 12% men. This reality has a difficult explanation.

The contingency tables of those who score 4 or 5, compared to the variables sex, type of center, children and teaching experience, are as follows (Table 4):

The proportion of men who score high (4 or 5) is higher, with a *p*-value = 0.02053 < 0.05, although, with such a tight *p*-value, the result is inconclusive. 

The contrast of proportions gives a *p*-value = 0.7532 > 0.05. Consequently, the proportion of teachers who score high (4 or 5) is the same in subsidized and public schools. 

The contrast of proportions of those who score 4 or 5 to contrast if the proportion among those who have children is higher gives a *p*-value = 0.03624 < 0.05. Therefore, it is higher among those who have children, although with an inconclusive result (very tight *p*-value).

The *p*-value = 0.3390 for the independence contrast (Fisher) indicates that the proportion of those who score 4 or 5 is similar according to teaching experience.

The independence contrast (Fisher) for the variables age and high scores (4 or 5) gives us a *p*-value = 0.02939. So, there is dependence between both variables. It seems that teachers over the age of 40 tend to give higher scores.

**Figure 8:**
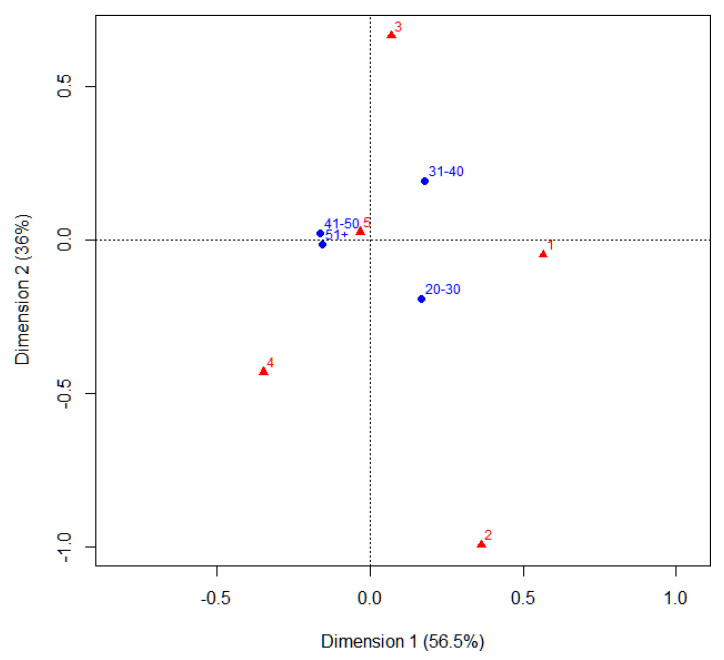
Symmetrical map of the answers, based on age.

If the dependency relationship between the variables age and rejection of prejudices is analyzed, it is observed how the contrast of independence (Fisher) gives a *p*-value = 0.03183 < 0.05. Therefore, both variables are dependent. The contingency table is as follows (Table 5):

Once the dependency relationship between the two variables has been established, it is interesting to know what this relationship is like. The answer to that question lies in correspondence analysis, which is used to represent possible associations between variables (factors) and to determine if it is possible to observe patterns. The distances between the different categories indicate the greater or lesser relationship between them; the relationships between the elements are displayed as distances in a graph (Figure 8).

Correspondence analysis shows that a score of 5 is associated with the highest age (over 41 years).

**Figure 9:**
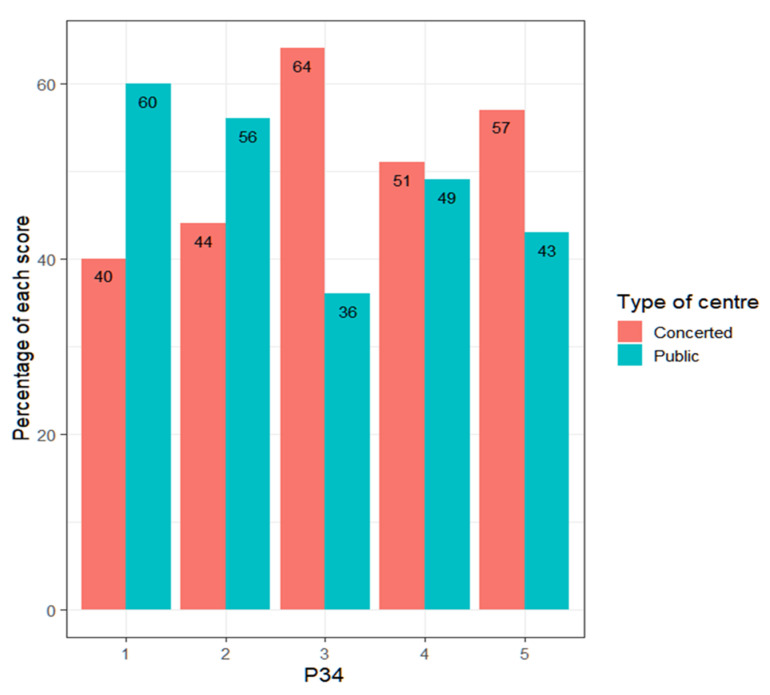
Percentage of each score according to the type of center.

Regarding the commitment of teachers to the personal and cultural management of students—which, again, is an indicator of respect for the students—it is observed how there are no major discrepancies depending on the type of center (Figure 9). 

The Wilcoxon test gives a *p*-value = 0.3149, confirming the visual impression. The distributions of the scores are similar. All teachers have a high commitment to the construction of the personal and cultural identity of their students, despite the difficulties that may arise. In addition, the number of teachers who score 4 or 5 depending on the type of center (contrast of proportions *p*-value = 0.6715) reinforces this idea (Table 6).

(c) Teaching vocation–self-esteem

In this section, it is observed how teachers who like their work tend to promote the self-esteem of their students. This denotes a concern for the emotional development of their students. Although the existence of this linear relationship is observed (ANOVA for linear regression, *p*-value = 0.006 < 0.05), this is not very intense: R2 = 0.018. For nonparametric correlations, the results are similar. The Kendall rank correlation coefficient (Kendall’s coefficient τ) of value τ = 0.175 is significant with a *p*-value = 0.001 < 0.05. Spearman’s rank correlation coefficient (Spearman’s ρ) of value ρ = 0.177 is significant (*p*-value = 0.001 < 0.05). The bubble graph of the scores, as well as the line adjusted to the point cloud using least squares, attest to this.


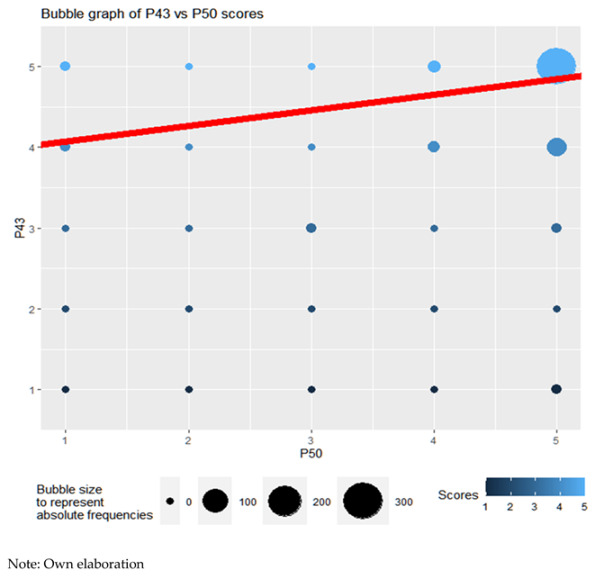

**Hypothesis** **2.** *Classroom management presents significant differences with respect to the emotional competences of teachers depending on professional and sociodemographic variables (sex, age, professional experience, type of center in which they work, whether they have children and teaching vocation)*.

(a) Conflict management

The way of managing conflicts by teachers has a high emotional charge within the educational process. Analyzing the preparation of teachers to resolve such conflicts in an appropriate way—taking into account the emotional aspects—falls within their professional competence. 

**Figure 10:**
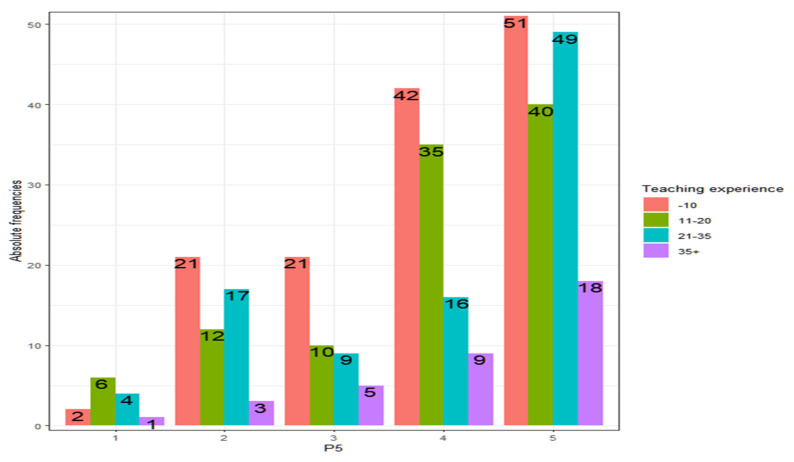
Absolute frequencies of responses vs. teaching experience.

As shown in Figure 10, all teachers score similarly in terms of their ability to deal with conflicts, regardless of their teaching experience (Kruskal–Wallis, *p*-value = 0.4863 > α = 0.05).

It should be noted that the number of teachers who believe they are not prepared to manage conflicts in the classroom is remarkable (Table 7).

These percentages of teachers who feel unprepared do not depend on sex (contrast of proportions, *p*-value = 0.9753), nor on whether they have children (contrast of proportions, *p*-value = 0.5505), on the type of center (contrast of proportions, *p*-value = 0.7839), on experience (contrast of proportions, *p*-value = 0.4920) or age (contrast of proportions, *p*-value = 0.9227).

(b) Methodological aspects

Creating learning environments for listening to students improves academic performance and the quality of interpersonal relationships. Both pedagogical initiatives have a high emotional depth and generate motivation and a desire to learn.

**Figure 11:**
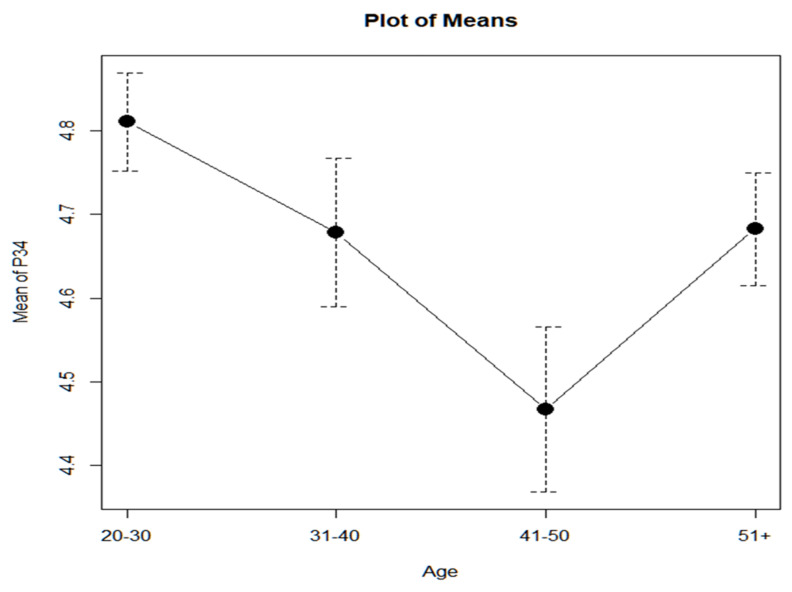
Graph of means by age.

Analyzing the results, it is observed that younger teachers (20–30 years old) are the ones who are most concerned with creating learning environments that stimulate work in the classroom and attention to diversity, mainly with respect to the group of teachers between 41–50 years old (Figure 11). As there is a case in which the variance of the groups with a center in the median, according to age, is not homogeneous (Levene with center in the median, *p*-value = 0.02509 < 0.05), we opt for a one-factor ANOVA for the assumption that the variances are not equal (Welch test).

(a) For this case, the one-way ANOVA *p*-value (age), not assuming equal variances (Welch test), gives us a *p*-value = 0.0251 < 0.05. Therefore, there are significant differences between the groups.
(b) Post hoc contrasts indicate that the highest average score is obtained by the 20–30 age group and that the 41–50 age group obtains the minimum average score, with the difference between the two groups being significant.
(c) The differences between the rest of the age groups are not significant.

Although the Kruskal–Wallis test is not recommended, it is convenient in this case to see if this test (with the corresponding post hoc contrasts) confirms the above results (Table 8):

(a) The Kruskal–Wallis test with a *p*-value = 0.002005 < 0.05 rejects the null hypothesis that the populations from which the samples have been extracted are equidistributed.
(b) Post hoc contrasts using Wilcoxon to compare each pair of age groups and adjusting the *p*-value using the Holm method give a similar result.

From the table, it can be deduced that the only significant difference occurs between age groups 20–30 vs. 41–50.

If the high responses (4 or 5) of teachers under 30 years of age compared to those over 30 years of age are analyzed, it is observed that the *p*-value for the contrast of proportions is as follows: *p*-value = 0.02477 < 0.05. It is accepted, therefore, that younger people score higher than older people, but with such a tight *p*-value, the decision is not conclusive (Table 9). The proportion of teachers who score high (4 or 5) is 95% CI = (90.24, 95.40), which shows that most feel committed to attention to diversity.

On the other hand, regarding the predisposition to listen to students and let them express their ideas and opinions, it is observed how teachers are the ones who listen to them the most (Wilcoxon, *p*-value = 0.1717) (Table 10).

**Figure 12:**
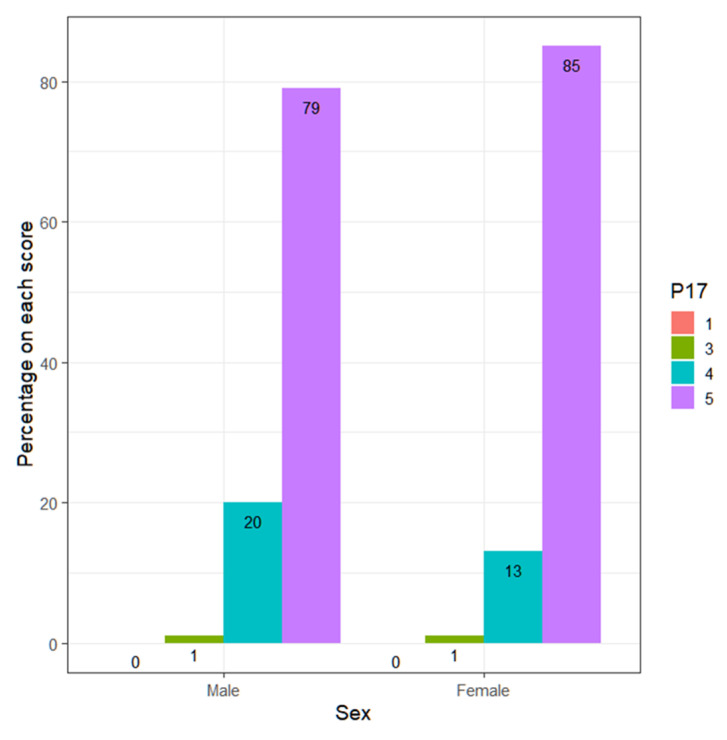
Percentage by sex of each of the responses.

It is observed that 78.9% of men score 5, while 85.1% of women score 5. Meanwhile, 20.0% of men score 4, while women score 13.4% (Figure 12). 

The teachers who score high (4 or 5), according to sex, are (Table 11):

The Fisher test to contrast the equality of proportions between both groups gives a *p*-value = 0.9999 > 0.05. Therefore, it cannot be denied that the proportion of teachers who score high (4 or 5) is the same between both sexes. The confidence interval for those who score high, regardless of sex, is as follows: CI95%= (96.9, 99.4).

(c) Vocation–conflict management

The results show how teachers who like their work tend, slightly, to prepare more to manage conflicts well, although this relationship is not very marked, since the correlation coefficients τ of Kendall (τ = 0.018) and ρ of Spearman (*ρ* = 0.112) are only slightly positive. Moreover, with such tight *p*-values, the decision on their significance is inconclusive. The bubble graph of the scores, as well as the line adjusted to the point cloud using least squares, prove it.


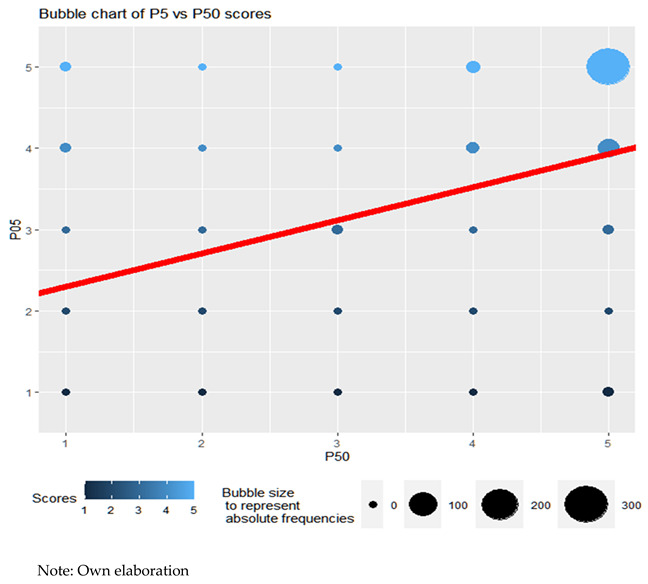


**Figure 13:**
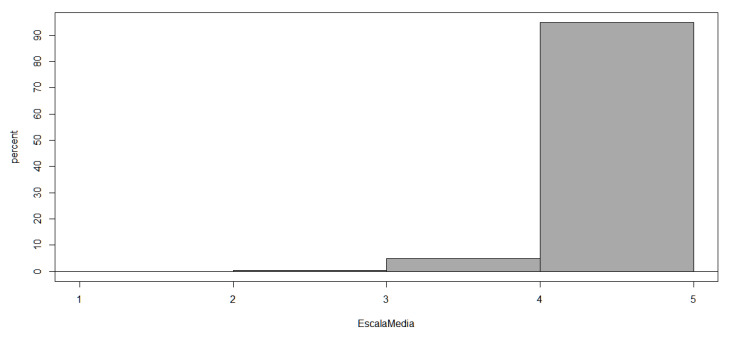
Average overall score of teachers’ emotional competencies.

To conclude the Results section, we wanted to answer the question posed at the beginning of this article: Do primary school teachers in the city of Valencia feel that they have adequate emotional competencies to deal with the emotional problems caused by COVID-19 among students? The following figure (Figure 13) clearly shows how teachers perceive themselves in terms of their emotional competence (4.6 out of 5):

## 5. Discussion

The aim of this research is to analyze the emotional competencies of primary school teachers in relation to two hypotheses. The first hypothesis, Hypothesis 1, which states whether the personal characteristics and emotional competencies of teachers interfere in student management, as a function of sociodemographic and professional variables, is not confirmed. There are no differences with respect to age, years of experience, the type of center in which they work, whether they have children or not, and in terms of teaching vocation. Differences are only found with respect to the sex variable. In this study, female teachers show a greater tendency to interpret and respond to the emotional demands of students. This result coincides with those of Molero et al. [63], Furham [64] and Stamatopoulou et al. [65], where it is the female student teachers who show better management of emotional situations. In the same line, it also coincides with the studies of López-Lujan and Sanz [37]; Pena, Rey and Extremera [66]; Llorent et al. [30]; Llorent and Núñez-Flores [29]; Molina et al. [31]; and Palomera et al. [67], who affirm that female teachers project themselves with more ability to attend and perceive students’ emotions. A study by Avissar [35] maintains that male teachers present greater difficulties in managing students’ emotions.

However, in the present study, the differences with respect to gender are not tangentially affirmative of this hypothesis, since, being a self-report, all teachers report respecting students’ emotions as well as indicate that they tend to foster students’ self-esteem. In this sense, the scientific literature advocates that all teachers become “actors of coherence” [68] (p.107). The adequate development of emotional competence among teachers improves the respectful teacher–student relationship [69,70,71], educational practice [43] and educates the civic aspect [72,73]. Similarly, all teachers show a high commitment to the construction of their students’ personal and cultural identity, despite the difficulties that may arise. In this sense, Jordán and Codena [74] agree on how the emotional aspect of teachers influences this construction, and a study with future teachers highlights that what concerns them most is to favor the personal growth and development of their students [75].

Regarding Hypothesis 2, which asks whether classroom management presents significant differences with respect to teachers’ emotional competencies as a function of professional and sociodemographic variables, in this study, the results indicate that there are no differences in terms of gender, age, professional experience, the type of center in which they work, whether they have children or their teaching vocation. Therefore, the hypothesis is not confirmed either. 

This conclusion coincides with studies such as those by Lozano et al. [76], Barrientos and Pericacho [43] and Keefer et al. [77], which indicate that all teachers are aware of the importance of the adequate emotional management of the classroom to manage students’ social–emotional competencies and make an effort to do so. However, they are not in line with the results obtained by Cuesta and Azcárate [78], Martínez-Saura et al. [32] and García-Domingo [33], who in their research find significant differences according to age and professional experience in classroom management, especially in relation to the management of attention to student diversity. Nor do our results coincide with those obtained in the macro study by Hattie [79], who affirms that teachers do not listen to their students, or with that carried out by Hattie and Yates [80], who confirm this conclusion and indicate that teachers’ lack of empathy distances them emotionally from students. Therefore, this result does not coincide with the self-perception presented in the present study, in which it is indicated that they listen to and attend to the emotional demands of the students.

It should be noted that this study coincides with many other studies carried out via self-reporting, which indicate that, although the results usually show differences with respect to gender on emotional management in the classroom, this conclusion presents an important bias, since all teachers in general perceive themselves as adequately being able to manage their students’ emotions. This high self-perception of teachers’ emotional competence is also reflected in the studies of Llorent and Núñez-Flores [29] and Lira et al. [81]. In contrast, there are studies in which teachers perceive themselves as having simply adequate competence [33,34] and demand more training [82].

These results have a clear implication from a practical point of view. All the teachers evaluated, regardless of gender, age, professional experience, type of center or whether they have children, show a high perception of their emotional competence as teachers. These results are very positive, since student and classroom management is not conditioned by any sociodemographic variable. Therefore, these results reaffirm that the educational system presents high levels of equity, equal opportunities and quality in all educational contexts and realities.

## 6. Conclusions

Primary school teachers in the city of Valencia perceive themselves as having a very high emotional competence, in all its dimensions. If we consider the hypotheses put forward in this research, we observe the following:

Hypothesis 1: “The personal characteristics and emotional competences of teachers interfere in the management of students, finding differences depending on sociodemographic and professional variables (sex, age, professional experience, type of center in which they work, if they have children and teaching vocation)”, is not confirmed. In general terms, personal and professional characteristics do not interfere with students’ emotional management. All teachers feel competent or very competent in the interpretation of emotions, in respect for the student, in the construction of their personal and cultural identity and in the promotion of self-esteem.

Only a few small differences are found. It is the teachers who feel more able to observe and interpret the emotions and/or feelings of their students. Male teachers more strongly reject any type of prejudice, racism or discrimination that may occur with respect to a student. The percentage of teachers who say they do not reject prejudice, discrimination and racism is worrying. Finally, it is the teachers with more vocation who promote the self-esteem of the students the most. 

Hypothesis 2: “Classroom management presents significant differences with respect to the emotional competences of teachers depending on the professional and sociodemographic variables (sex, age, professional experience, type of center in which they work, if they have children and teaching vocation)”, is also not confirmed. Classroom management does not present significant differences depending on the variables analyzed.

Only small differences are observed: It is the youngest teachers who show the most sensitivity when it comes to creating learning environments that cater to diversity. In the same way, teachers with more vocation are those who claim to feel more qualified to resolve classroom conflicts. There is concern about the percentage of teachers who say they do not feel prepared to manage classroom conflicts.

The hypotheses cannot be fully confirmed. Only some small aspects establish significant differences according to sociodemographic variables.

As for the possible limitations of this research, these lie in the sample, since the results are limited to the city of Valencia, so they cannot be generalized to other realities. Another limitation lies in the structure of the research, since it is a self-perception questionnaire, and the scores tend to be very high. It would be interesting to contrast these results with the opinions of the students.

As for future lines of research, these revolve around the need to implement this study in other cities and/or autonomous communities, to present an X-ray of national teachers around their emotional competences and, thus, to be able to implement continuous training programs. The aim is also to analyze the emotional competencies of teachers at all levels of the educational system—secondary education, baccalaureate and training cycles—since the psycho-emotional impact and mental health problems among young students are also very important.

## Figures and Tables

**Table 1:** Sample composition.

Sex	Women	74.4%
	Men	25.6%
Age	Between 20–30 years	24.3%
	Between 31–40 years	23.5%
	Between 41–50 years	24.3%
	More than 50 years	28%
Ownership of the center	Public schools	43.9%
	Subsidised centres	56.1%
Teaching experience	Less than 10 years	36.9%
	Between 11 and 20 years old	27.8%
	Between 21 and 35 years old	25.6%
	More than 35 years	9.7%
Children	If they have children	60.4%
	They have no children	39.6%

**Table 2:** Items regarding emotional competence and character.

Questions	Character
5. I am not prepared to resolve or manage conflicts in the classroom or in the center	CMP
17. I listen to the questions and questions that students ask me	CMM
26. I generate an environment in which personal and cultural differences are perceived as mutual enrichment	CMM
27. I observe and interpret students’ emotions and/or feelings	CMM
28. It is difficult for me to relate to classmates, students and families	CMP
29. It is not my role to properly observe and interpret the possible risk behaviors of students (addictions, abuse, …)	CMP
31. I feel responsible for student learning	CMM
34. I am not committed to managing the cultural and personal diversity of students	CMP
37. I do not feel committed to the collaboration with the Management Team of the Center	CMP
42. Respect for the student	CMM
43. Promoting students’ self-esteem	CMM
45. I reject prejudice, racism and discrimination	CMM
50. I don’t like the teaching profession	CMP

Note: CMM stands for “cuanto más mejor” in Spanish (“the higher, the better” in English); CMP stands for “cuanto más peor” in Spanish (“the higher, the worse” in English).

**Table 3:** Proportion of teachers who score high against sociodemographic variables.

Sex	Score 4 or 5	Score 1, 2 or 3	% Score 4 or 5	*p*–Fisher’s Test Value	Conclusion
Man	94	1	98.9%	0.04826 < 0.05	The proportion of those who score high is higher among women than among men. Inconclusive decision.
Woman	274	2	99.3%		
**Type of center**	**Score 4 or 5**	**Score 1 2 or 3**	**% score 4 or 5**		
Concerted	207	1	99.5%	0.8484 > 0.05	The proportion of those who score high is similar between public and private schools.
Public	161	2	98.8%		
**Children**	**Score 4 or 5**	**Score 1 2 or 3**	**% score 4 or 5**		
No	146	1	99.3%	0.05285 > 0.05	The proportion of those who score high is similar among those who have children or not. Inconclusive decision.
Yes	222	2	99.1%		
**Experience teacher**	**Score 4 or 5**	**Score 1 2 or 3**	**% score 4 or 5**		
−10	136	1	99.3%	0.3390 > 0.05	The proportion of those who score high is similar according to teaching experience.
11–20	102	1	99.0%		
21–35	95	0	100%		
35+	35	1	97.2%		
**Age**	**Score 4 or 5**	**Score 1 2 or 3**	**% score 4 or 5**		
20–30	90	0	100%	0.02939 < 0.05	The proportion of those who score high differs according to age. The decision is inconclusive.
31–40	86	1	98.9%		
41–50	89	1	98.9%		
51+	103	1	99.0%		

**Table 4:** The contingency tables for teachers scoring 4 or 5, compared to the variables gender, type of center, children, and teaching experience.

(a) For sex			
Sex	Score 4 or 5	Score 1, 2 or 3	% Score 4 or 5
Man	92	3	96.8%
Woman	249	27	90.2%
**(b) For type of center**			
**Type of** **Center**	**Score 4 or 5**	**Score 1, 2 or 3**	**% Score 4 or 5**
Concerted	192	16	92.3%
Public	149	14	91.4%
**(c) For children**			
**Children**	**Score 4 or 5**	**Score 1, 2 or 3**	**% Score 4 or 5**
No	130	17	88.4%
Yes	211	13	94.2%
**(d) For teaching experience**			
**Teaching** **Experience**	**Score 4 or 5**	**Score 1 2 or 3**	**% Score 4 or 5**
−10	123	14	89.8%
11–20	93	10	90.3%
21–35	91	4	95.8%
35+	34	2	94.5%
**(e) To age**			
**Age**	**Score 4 or 5**	**Score 1 2 or 3**	**% Score 4 or 5**
20–30	78	12	86.7%
31–40	77	10	88.5%
41–50	87	3	96.7%
51+	99	5	95.2%

**Table 5:** Absolute frequencies and percentage of each score.

	Ratings for P45				
Age	1	2	3	4	5
20–30	10 (40%)	2 (66.7%)	0 (0.0%)	4 (30.8%)	74 (22.6%)
31–40	9 (36%)	0 (0.0%)	1 (50%)	0 (0.0%)	77 (23.5%)
41–50	3 (12%)	0 (0.0%)	0 (0.0%)	4 (30.8%)	83 (25.3%)
50+	3 (12%)	1 (33.3%)	1 (50%)	5 (38.5%)	94 (28.7%)
Total	25 (100%)	3 (100%)	2 (100%)	13 (100%)	328 (100%)

**Table 6:** Teachers who score high according to the type of center.

Center Type	Score 4 or 5	Score 1 2 or 3	% Score 4 or 5
Concerted	195	13	93.8
Public	151	12	92.6

**Table 7:** Percentage of teachers scoring low and Teaching experience.

Teaching Experience	Less than 10 Years	Between 11–20	Between 21–35	More than 35 Years
% scoring 1 or 2	16.8%	17.5%	22.1%	11.1%

**Table 8:** Pairwise comparisons using Wilcoxon’s rank sum test with continuity correction.

Age			
	20–30	31–40	41–50
31–40	0.839	-	-
41–50	0.013	0.146	-
51+	0.452	0.839	0.297

*p* value adjustment method: Holm.

**Table 9:** High scores for ages over 30 and under 30.

Age	Score 4 or 5 on P34	Score 1, 2 or 3 in P34	% Scoring 4 or 5
Under 30	88	2	97.8%
Over 30	258	23	91.8%

**Table 10:** Absolute frequencies and percentage of each score according to sex.

Table of Frequencies						
Sex	1	2	3	4	5	Total
Man	0	0	1	19	75	95
Woman	1	0	3	37	235	276
**Percentage**						
**Sex**	**1**	**2**	**3**	**4**	**5**	**Total**
Man	0.0%	0.0%	1.1%	20.0%	78.9%	100%
Woman	0.4%	0.0%	1.1%	13.4%	85.1%	100%

**Table 11:** High scores by sex.

Sex	Score 4 or 5	Score 1, 2 or 3	% Score 4 or 5
Man	94	1	98.9%
Woman	272	4	98.6%
